# Oral health professional intervention and child physical abuse—European legal approach

**DOI:** 10.1093/fsr/owad042

**Published:** 2023-12-06

**Authors:** Ana Corte-Real, Pedro Armelim Almiro, Mafalda Silva, Tiago Nunes, João Abreu, Carla Carreira, Duarte Nuno Vieira

**Affiliations:** Faculty of Medicine, Laboratory of Forensic Dentistry, University of Coimbra, Coimbra, Portugal; Autonomous University of Lisbon, Centro de Investigação em Psicologia, Lisboa, Portugal; Faculty of Medicine, Laboratory of Forensic Dentistry, University of Coimbra, Coimbra, Portugal; Faculty of Medicine, Laboratory of Forensic Dentistry, University of Coimbra, Coimbra, Portugal; Faculty of Medicine, Laboratory of Forensic Dentistry, University of Coimbra, Coimbra, Portugal; Instituto Nacional de Medicina Legal e Ciências Forenses, Coimbra, Portugal; Faculty of Medicine, Laboratory of Forensic Dentistry, University of Coimbra, Coimbra, Portugal

**Keywords:** child abuse, aetiology, family issues and mediators, intervention

## Abstract

Physical violence against children and adolescents is an issue of Global Public Health. This study aims to identify traumatic injuries and the medicolegal temporary framework of the victim’s profile in the European legal approach. Participants and setting include the following: the clinical reports of a Portuguese European Clinical Academic Center database were analysed. An observational and prospective cohort study was performed. A descriptive analysis of the variables was conducted, considering gender, bimodal age groups, place of residence, offender data, place of occurrence, aetiology, localization, type of injuries, personal injury assessment by *Quantum doloris*, and injury time. The statistical analysis was performed by Spearman’s rho and Kendall’s tau-b correlation tests, Pearson’s chi-square test of independence (χ^2^), and Mann–Whitney and Kruskal–Wallis nonparametric tests (*P* < 0.05). The relationship between age groups and the place of occurrence was statistically significant (*P* = 0.001). Orofacial and nonorofacial injuries were related (*P* = 0.035). The General Data Protection Regulation is not a barrier to the treatment and sharing of justified data but a framework for safeguarding individuals’ fundamental rights, including the Right to Health. Meticulous reporting of the clinical situation involves the victim, the occurrence, and the potential offender.

**Key points:**

- An oral health professional’s notification of the event is a fundamental step of the judicial process.
- The timeline is the core of traumatic injury assessment in a child physical abuse scenario.
- Medicolegal evaluation impacts disability prevention, imputability, and notification of the crime.

## Introduction

The World Health Organization (WHO) defines violence as the intentional use of physical force or power against oneself (intrapersonal), another person (interpersonal), or a group or community (collective), which can or does result in injury, death, psychological harm, maldevelopment, or deprivation [1–3]. Interpersonal violence is the 19th aetiology of morbidity and mortality, emerging as a global health issue [3]. Violence types are sexual, psychological, and physical, and focus on the age range [3]. Physical violence against children and adolescents (PVCA) can result in trauma injuries in children under 18 years old [3], due to actions such as punching, beating, kicking, biting, burning, shaking, or other harmful methods [3]. Physical abuse can potentially result from corporal punishment [4], where the parent or caretaker may not have intended to hurt the child; rather, the injury may have resulted from excessive discipline or physical punishment. Physical abuse can encompass bullying [5] as a form of child abuse in school. Bullying, as defined from the educator’s perspective according to Olweus (1993), involves aggressive behaviour that includes unwanted or harmful actions repeated over time and an imbalance of power or strength. From the student’s perspective, it can range from “when someone is mean” to physical and sexual harassment [6].

Physical traumatic injury results from an external cause related to the interaction of the body with energy (mechanical, thermal, electrical, chemical, or radiant or due to extreme pressure) in an amount or at a transfer rate that exceeds physical or physiological tolerance [7]. The coding and classification of injuries assign the victim to the appropriate profile.

Anatomically, the craniofacial region is one of the most vulnerable corporal regions to traumatic injury [8]. According to the American College of Surgeons, the head is the second region with the highest incidence of traumatic injuries, and the face is the fourth region [9, 10]. Clinical observation and detailed records on the orofacial region are essential in diagnosing and identifying physical violence as a step in the legal process.

In the context of human rights, the definition of the injured profile allows the medicolegal assessment of personal injuries [11, 12]. Medicolegal assessment of injured victims should consider temporary and permanent disabilities [12], considering the temporal duration of the recovery process and the evidence of deficits in bodily integrity or sequela. Long recovery times and complex facial rehabilitations are related to disability severity [13, 14]. The aftermath of a traumatic event in children and adolescents impacts the victim’s development, emotional behaviour, school performance, and social relationship. Access to timely and effective care involves early diagnosis and treatment resources that help reduce disability.

Assessment of law and regulation by healthcare professionals is crucial. Medical and personal data management must comply with national and international guidelines. The General Data Protection Regulation (GDPR), signed in 2016 and applied in May 2018, addressed the privacy and protection of personal data for all individuals in the European Union and European Economic Area [15]. Because it is a regulation and not a directive, there is no need for additional legislation to be passed by member states [15].

This study aimed to highlight the mandatory role of oral health professionals in screening for physical abuse as a legal procedure for children and adolescents’ human rights.

## Material and methods

An observational and prospective cohort study was performed in the Clinical Academic Center of Coimbra (CACC), following the Strengthening the Reporting of Observational Studies in Epidemiology methodology. The CACC is a consortium between the University and Hospital Center of Coimbra and the Faculty of Medicine at the University of Coimbra, Portugal.

This study included electronic health records of individuals aged 0–16 years with oral and auto-defense injuries related to trauma events and a history of interpersonal violence between January 2020 and May 2022. Medical reports with nontraumatic injury diagnoses (e.g. genetic diseases), sexual violence, and neglect cases were excluded. Written informed consent was provided in compliance with the Faculty of Medicine Ethics Committee (CE-066/2022). The study was conducted in accordance with the Declaration of Helsinki and approved by the Ethics Committee of the Faculty of Medicine (CE-048/2017).

A dental examination of the victim was conducted by a team dentist in collaboration with a forensic odontologist from the Laboratory of Forensic Dentistry at the University of Coimbra, Portugal. The research team, with up to 15 years of experience in medicolegal expertise, analysed the consistency of history and clinical data.

The reports that met the inclusion criteria were reviewed and interpreted according to gender, age, place of occurrence, anatomy of the injury, injury diagnosis, injury time and severity, relationship with the alleged offender (if any), and the time elapsed between the aggression and the examination.

According to age range, two groups were considered in a bimodal distribution: less than or equal to 12 years old (G1) and 13 years and older (G2) [16].

Regarding the place of occurrence, in accordance with WHO guidelines [1], the sample was divided into four groups: home, school, public place, and sports practice place.

According to the anatomy of the injury, 10 body regions were considered [9]. As a subdivision of the face, the orofacial region corresponds to the lower one-third of the face, between *subnasale* and *menton* points [17].

The injury diagnosis performed according to the International Code of Diseases (ICD-11) [7] allowed categorization of the sample into five diagnosis groups: (i) superficial and orofacial (codification for intraoral injury and extraoral injury), (ii) superficial and no orofacial, (iii) bone fractures, (iv) dental trauma (cod for hard and periodontal injuries), and (v) temporomandibular trauma.

The duration of the illness, referred to as “injury time”, was studied regarding the medical appointments and limitations in activities. This period ended with the discharge date, corresponding to the end of rehabilitation or the healing period of the injury, or the definition of sequela in the medicolegal context.

Statistical analysis was performed using IBM SPSS® Statistics (version 26; IBM Corp., Chicago, IL, USA). Spearman’s rho and Kendall’s tau-b correlation tests were used for variable associations (continuous variables). For categorical variables, Pearson’s chi-square test of independence (χ^2^) was also used, and the Mann–Whitney and Kruskal–Wallis nonparametric tests were used to compare groups. The statistical significance level was set at *P* < 0.05.

## Results

The sample (*N* = 30) included victims aged between 6 and 16 years (mean age = 9.6 ± 3.1 years), with 80% in the age range of 0–12 years (G1) and 20% in the age range of 13–16 years (G2). In both groups, males were more frequent (G1 = 58.3% and G2 = 66.7%). Regarding the area of residence, 79.2% lived in rural areas in G1 and 83.3% in G2.

### Offender data

In 76.7% (*n* = 23) of the cases, one offender was involved: parent (*n* = 18, 60.0%), aunt (*n* = 2, 6.7%), grandmother (*n* = 2, 6.7%), and colleague (*n* = 1, 3.3%). In 23.3% (*n* = 7) of situations, there were two (*n* = 3, 10%) or more (*n* = 4, 13.3%) offenders, often identified as colleagues within a school context (bullying). Alcohol consumption was involved in 30.0% (*n* = 10) of the events.

### Place of occurrence

**Figure 1:**
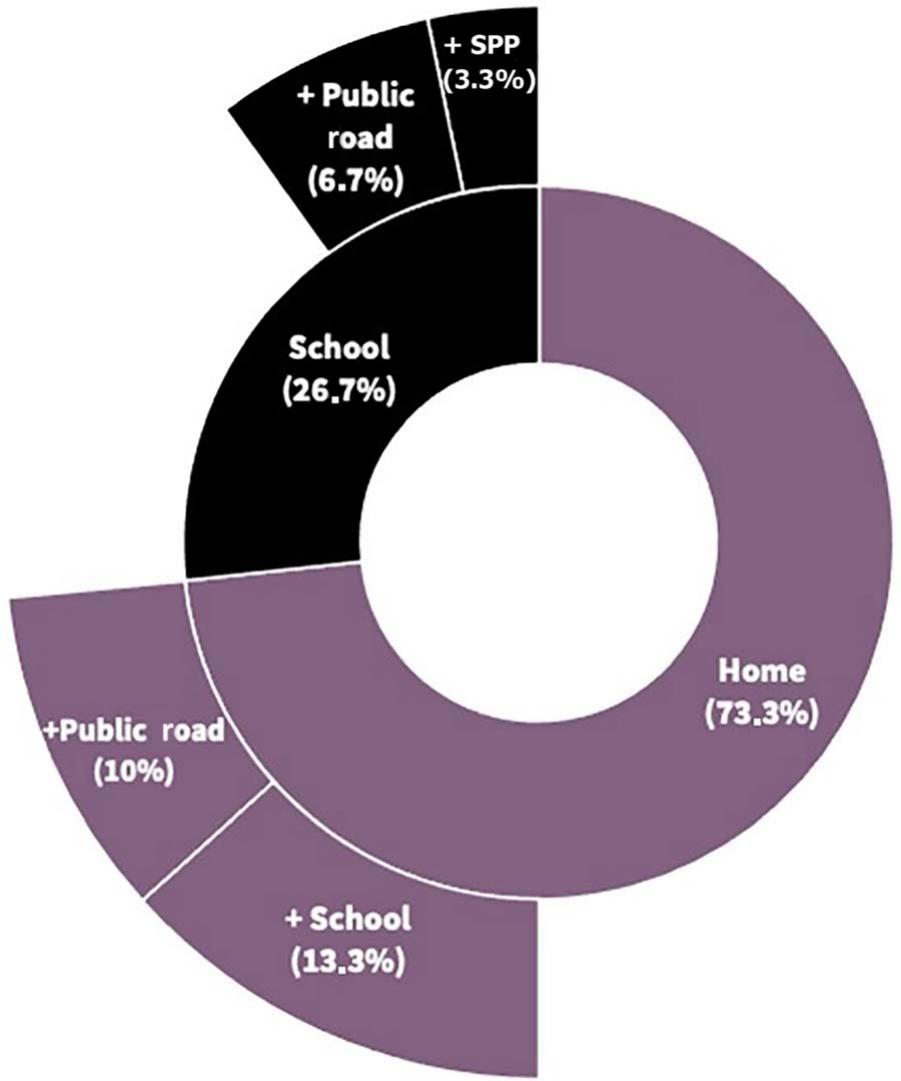
Places of occurrence in two-ring graphic (*N* = 30). SPP: sports practice place.

The home served as the place of occurrence for 73.3% (*n* = 22) of the sample, either as an isolated place (*n* = 15, 50.0%), in association with a school environment (*n* = 4, 13.3%), or associated with a public road (*n* = 3, 10.0%). The school emerged as the central place for violence in 26.7% (*n* = 8) of the cases, either as an isolated place (*n* = 5, 16.7%), in association with a public road (*n* = 2, 6.7%), or a sports practice place (*n* = 1, 3.3%) (Figure 1).

### Aetiology, localization, and type of injuries

**Figure 2:**
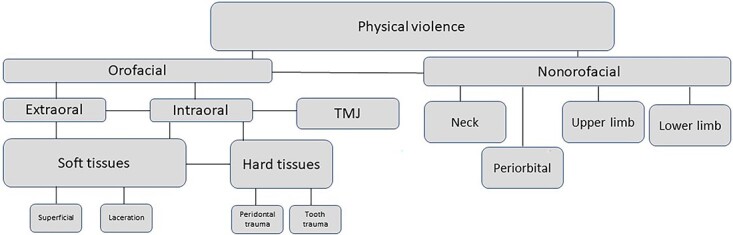
Injury patterns concerning anatomical topography. TMJ: temporomandibular joint.

Self-defense injuries were documented in 10.0% (*n* = 3) of the sample. The patterns of injuries (Figure 2) were identified: intraoral (*n* = 7, 23.3%), extraoral (*n* = 3, 10.0%), and both lesions (*n* = 20, 66.7%).

Intraoral soft tissue injuries were identified primarily as lacerations (Na01.3, in oral cavity XA1WN1) on the inner surface of the lips or the frenulum, labial, lingual, and buccal mucosa. Moreover, these injuries were identified as superficial (Na00.4). Intraoral injuries due to dental trauma (36.7%) (Na0D) included enamel (Na0D.01) and crown-root fractures (Na0D.05) in upper central incisors teeth. Intraoral lesions at the level of periodontal tissues involved concussions, lateral dislocations, and intrusive dislocations affecting upper central or lateral incisors. Temporomandibular joint (TMJ) injuries (10%) were diagnosed as disc compression (Na03.3), bilaterally (*n* = 2) and were associated with mouth opening reduction (*n* = 1) (SC0705 and SC0706). Extraoral soft tissue injuries (Na00.5) manifested as bruises, abrasions, and lacerations on the chin, face, and lips. No facial bone fractures were identified (Na02). Nonorofacial injuries (*n* = 29) occurred in the upper limbs (arm, forearm, and hands) (*n* = 25, 83.3%) as excoriations and bruises. Injuries of the neck (*n* = 22, 73.3%) occurred as bruises in the retro auricular or lateral area, lower limbs (*n* = 5, 16.7%), and periorbital region (*n* = 3, 10.0%).

### Personal injury assessment

**Figure 3:**
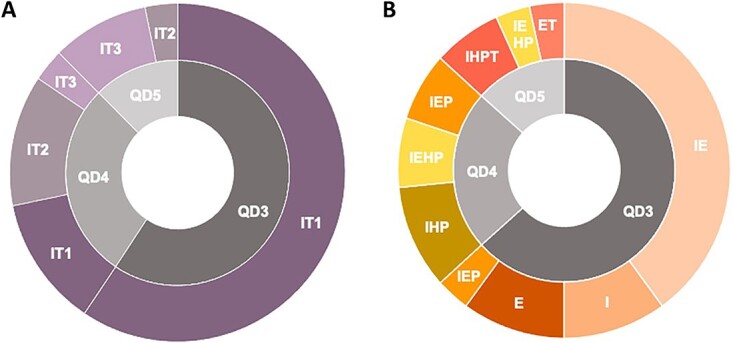
The two-ring graphic presents the temporary injury evaluation (*N* = 30). (A) *Q. doloris* and injury time or temporary incapacity; (B) *Q. doloris* and orofacial injuries. IT1: minor degree of injury time; IT2: average degree of injury time; IT3: major degree of injury time; QD3: *Q. doloris* grade 3/7; QD4: *Q. doloris* grade 4/7; QD5: *Q. doloris* grade 5/7; I: superficial intraoral injuries; E: superficial extraoral injuries; IE: superficial intraoral and extraoral injuries; IEP: superficial intraoral and extraoral periodontal tissue injuries; IHP: superficial intraoral, hard, and periodontal tissue injuries; IEHP: superficial intraoral and extraoral, hard, and periodontal tissue injuries; IHPT: superficial intraoral, hard, and periodontal tissues, and temporomandibular joint (TMJ) injuries; ET: superficial extraoral and TMJ injuries.

**Figure 4:**
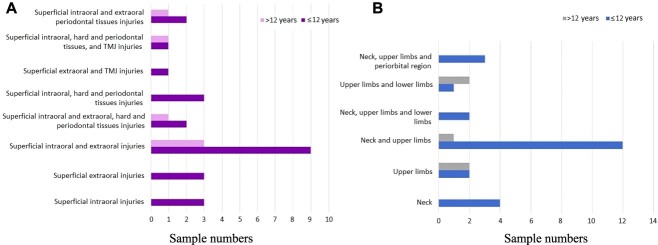
The bar graphic presents the descriptive analysis of the orofacial injury patterns (A) and nonorofacial injury patterns (B) and age groups (*N* = 30). TMJ: temporomandibular joint.

The healing time of injuries categorized the sample into three degrees (3/7, 4/7, and 5/7) of *Quantum doloris* (Figures 3 and 4) among the seven degrees. The comparison between *Q. doloris* and the injury time (minor = up to 100 days, medium = from 100 to 200 days and major = over 200 days) is shown in Figure 3A. The descriptive analysis between *Q. doloris* and orofacial injuries is presented in Figure 3B. Minor values of *Q. doloris* corresponded to minor injury times.

### Independence tests

The relationship between sex and orofacial and nonorofacial injuries was analysed using the χ^2^ test and was not statistically significant. The study of the relationship conducted by the χ^2^ test between age groups and the place of occurrence was statistically significant (χ^2^ = 21.875, df = 5, *P* = 0.001) according to Cramer’s V = 0.85 (large size effect). The χ^2^ test also demonstrated that age groups and the degree of attachment of the aggressor to the victim are related variables (χ^2^ = 20.625, df = 5, *P* = 0.001) according to Cramer’s V = 0.83 (large size effect). The analyses of the relationships performed by the χ^2^ test between age groups and the place of occurrence with nonorofacial injuries were statistically significant (χ^2^ = 13.8014, df = 6, *P* = 0.032); (χ^2^ = 68.038, df = 30, *P* = 0.000), respectively; however, these relationships with orofacial lesions were not statistically significant.

The analysis of the relationship performed by the χ^2^ test between the number of aggressors and the functional and esthetic impairment was statistically significant for orofacial lesions (χ^2^ = 19.182, df = 8, *P* = 0.014) according to Cramer’s V = 0.57 (large size effect) and for nonorofacial lesions (χ^2^ = 19.1675, df = 10, *P* = 0.032) according to Cramer’s V = 0.57 (large size effect).

The relationship between orofacial and nonorofacial injuries was statistically significant (χ^2^ = 60.096, df = 42, *P* = 0.035, *P* < 0.05) with Cramer’s V = 0.58 (large size effect).

### Associations and comparisons

In orofacial injuries, the correlations between functional and esthetic impairment (FEA) with *Q. doloris* and injury time were statistically significant (Table 1). However, identical analyses for nonorofacial lesions were not statistically significant.

**Table 1:** *Quantum doloris* and injury time following Spearman’s *rho* and Kendall’s tau-b tests.

	*Quantum doloris*	Injury time
Spearman’s *rho*	0.702	0.737
Kendall’s *tau-b*	0.654	0.687
*P*	0.000	0.000

Comparison for the place of residence (urban and rural), considering injury time (Mann–Whitney’s *U* = 12.00, *P* = 0.000), *Q. doloris* (Mann–Whitney’s *U* = 18.50, *P* = 0.001), and orofacial injuries (Mann–Whitney’s *U* = 28.50, *P* = 0.018), which are statistically significantly.

The comparison for the place of occurrence, considering injury time (Kruskal–Wallis’ *H* = 16.256, df = 5, *P* = 0.006) and *Q. doloris* (Kruskal–Wallis’ *H* = 13.186, df = 5, *P* = 0.022) was statistically significant. However, it was not statically significant for nonorofacial injuries (Kruskal–Wallis’ *H* = 10.842, df = 5, *P* = 0.050).

## Discussion

Focused on children and adolescents (PVCA), this study highlighted the intervention of health professionals as relevant actors in crime notification and medicolegal assessment.

The incidence of violence mainly occurs in men, following the literature [16, 18], although there is no statistically significant correlation between gender and the pattern of injuries, as suggested in the study by Cairns et al. [8].

Concerning the victim’s age, the average (9.63 ± 3.12) years was lower than a similar Portuguese study (13.37 ± 3.91) years [16]. In the present study, the victims were mainly under 12 years old, seeking interventive oral healthcare, without previous notification, compared with the age group 15–17 years old of the population evaluated in medicolegal issues after the notification procedure [16].

The victim’s age at the event time impacts their performance as an adult. It relates to developing language or communication skills to describe their condition and their cognitive ability to process the traumatic event [4, 8]. Health professionals should detail history data and its relation with clinical data. It should be carefully analysed to include the correct information in the legal process, as they are co-actors in the life pathway. Conflicting information (10%) or inconsistent data shared by the victim or their guardian may indicate a compromised interpersonal relationship that needs further analysis.

The data from the present study indicate the predominance of violence occurring at home (73.3%), are statistically significant, and are consistent with Cairns’ study [8] (53%) and global record data [10]. It can occur in association with a public place or school. In early childhood, parents significantly influence individual behaviours, and peers gain increased influence in adolescence [4]. Unfortunately, parents are potential aggressors in the age range from 0 to 12 years old, in the early stages of individual development under parental authority. The frequency of the traumatic event, homogeneous and consistent in the sample, can lead to its association as an outcome of corporal punishment [4], considering corporal punishment as a type of physical abuse. In this scope, health professionals should pay attention to items related to corporal punishment in the family environment, such as larger family structure, the quality of the parents’ romantic relationship, and marital status [4]. Social distancing measures emphasize parental stress over socioeconomic and psychosocial risk and its challenges in the COVID-19 pandemic. An increase in PVCA in this period was related to increased exposure of this vulnerable population to potential offenders and decreased interaction with notification actors.

In a bimodal age range criterion [8], above 13 years of age, the school emerges as the elective place of occurrence for the adolescent victim, which is statistically significant and aligns with general orofacial traumatology data [13, 19]. Violence in the school environment was related to several offenders (23.3%), specifically tied to a particular type of crime—bullying [5]. The ubiquitous nature of bullying, where students may perceive it as the norm; feelings of helplessness and vulnerability; a lack of self-reliance or personal responsibility in facing violence; and the shame associated with victimhood are factors contributing to bullying as an aetiology of violence in school [6]. Understanding bullying from the victim’s perspective is the first step to minimizing it and encouraging reporting [6]. The quality of the victim–health professional relationship serves as a crucial factor in encouraging violence notification, especially among older adolescents [6].

Public places also emerge as settings for violent incidents, aligning with the study by Vidal et al. [16], particularly among individuals aged 15 years and older (45.5%).

The familial connection between the victim and the offender, whether the father or the mother, is outstanding in the study, aligning with a United Nations Children’s Fund (UNICEF) report [20]. The relationship between the victim and the alleged offender holds significance in terms of personal data and legal issues, particularly concerning the minor age status [15]. In the European approach, the GDPR reiterates the need for consent from guardians or those with parental responsibility for individuals under the age of 16 years old. However, it is not a barrier to justified data processing and sharing. It provides a framework to ensure that such sharing is appropriate and prioritizes the safety and well-being of the child. National guidelines on good practices added information to the international regulation. The Data Protection Act 2018, published in the UK, explicitly states that professionals can share unique category data (e.g. medical data) without consent if it is done to safeguard children and individuals at risk. The Portuguese Data Protection Act (Law no. 58/2019, 8 August) states that it can only give consent for processing minors’ data after age 13 and is omissive about collecting and processing children’s data and adults at risk. In addition, health professionals are mandated to report risk situations and victim status under Art. 242 (Compulsory termination) of the Portuguese Constitution. Therefore, sharing information without informed consent lacks justification for each specific case. It should highlight keeping a record of the decisions performed and the reasons underlying those decisions: sharing or not sharing information, its content, with whom, and its purpose [15].

The data analysis emphasizes a standardized classification and codification of traumatic injuries, enabling the definition of the injured victim’s profile concerning the pattern of injuries in physical abuse (ICD11). The injury pattern is related to its physical location and type [8, 17]. The present study categorized injuries into 10 body regions, following the American report of 2016. The head is the second corporal area, and the face is the fourth corporal area of all traumatic injuries [9]. Previous studies focused on interpersonal violence have also highlighted the face and the head [9, 16, 18] due to access and anatomical vulnerability to physical impacts. In line with the recent systematic review, coexistent injuries included upper and lower limbs, emphasizing the offender’s intent of maltreatment action [18]. The orofacial area definition in the literature misses anatomical references for data analysis; according to Cairns et al. [8], it includes the head, face, neck, and mouth. It was also defined as having the face and head [21] or head and neck [22]. The orofacial region standardization through references of Arnett et al. [17] (planes and craniometric points) is mandatory for data comparison. The recent actualization of the ICD11 added the Andreasen Classification of dental trauma into Chapter 22 of ICD11.

The present study intended to correlate the individual’s clinical status with a victim profile. It recorded the individual clinical status associated with the injury’s patterns, making analysis more realistic about the victim profile. It became the outcomes’ discussion more complex because the literature examines each injury pattern individually, neither their relationship in association nor the surrounding factors.

Healthcare should consider an integrated corporal assessment, including orofacial and nonorofacial corporal areas, following its statistically significant correlation with the present data, overcoming the lack of this analysis in the bibliography available.

Nonorofacial injuries achieved statistical significance in their correlation with age range: superficial orofacial injuries with bruises in the neck and upper limbs (G2) and with bruises in the upper and lower limbs (G1). Interpersonal violence is reported as the third trauma aetiology in children and adolescent groups [13, 23], which should highlight its impact on the topographic characterization of injuries.

The victim’s disability is a relevant issue in individual autonomous development [13] and academic outcomes [24]. The impact of function and esthetic affectation in traumatic events, as injury consequences, can be analysed as *Q. doloris* and injury time within a temporary disability period [12]. The temporary disability evaluation of the sample justified the value of *Q. doloris* to the recovery of pain and the victim’s social affectation or social absence. The correlation between *Q. doloris* and injury time was statistically significant and justified by the type of injury (Figure 3). Superficial soft tissue injuries are related to the fast healing and rapid rehabilitation of functions [13]. In comparison, a long injury time is related to dental or TMJ trauma [13].

There was a statistically significant correlation between functional and esthetic affectation with the number of aggressors, meaning the predictability of the degree of affectation. The number of offenders can be related to the strength of the traumatic event and the physiological adaptation of biological tissues [13].

There was a statistically significant correlation between the place of residence, injuries, injury time, and *Q. doloris*. The rural place can be related to the lower efficiency and regulation of prevention measures and close environment of friends and family elements.

The craniofacial region is the intervention region for oral health professionals and is the most vulnerable and susceptible to traumatic injuries [13, 16]. This way, these professionals are at the forefront of detecting traumatic injuries and reporting crime. In the context of oral health consultations, the examination of the victim takes place within the first 8 days after the assault, while the medicolegal examination takes place, on average, within 14 days after the assault [13, 16]. The time factor is essential for the correct diagnosis of injuries and for medicolegal assessments, as well as for the notification of the crime and isolation of the victim.

As a pilot study, the present research analyzes the notification function of oral health professionals. The low sample rate could be biased although the physical violence can be related to cultural and national good practices. Overall, as a therapeutic procedure to overcome the injured condition, oral health professional activity complemented forensic dentists’ expertise in preventing physical abuse in children and adolescents at a younger age.

## Conclusion

Meticulous reporting of the clinical situation facilitates the identification and detailed organization of standardized data, the analysis of the imputability of the crime by its perpetrator, and the evaluation of temporary disability. The intervention of oral health professionals proves to be essential for minimizing PVCA and the medium- and long-term monitoring of the development of children and adolescents as victims of interpersonal physical violence.
